# Neuronal migration and protein kinases

**DOI:** 10.3389/fnins.2014.00458

**Published:** 2015-01-13

**Authors:** Toshio Ohshima

**Affiliations:** Laboratory for Molecular Brain Science, Department of Life Science and Medical Bioscience, Waseda UniversityTokyo, Japan

**Keywords:** protein phosphorylation, kinase, phosphatase, migration, cerebral cortex

## Abstract

The formation of the six-layered structure of the mammalian cortex via the inside-out pattern of neuronal migration is fundamental to neocortical functions. Extracellular cues such as Reelin induce intracellular signaling cascades through the protein phosphorylation. Migrating neurons also have intrinsic machineries to regulate cytoskeletal proteins and adhesion properties. Protein phosphorylation regulates these processes. Moreover, the balance between phosphorylation and dephosphorylation is modified by extracellular cues. Multipolar-bipolar transition, radial glia-guided locomotion and terminal translocation are critical steps of radial migration of cortical pyramidal neurons. Protein kinases such as Cyclin-dependent kinase 5 (Cdk5) and c-Jun N-terminal kinases (JNKs) involve these steps. In this review, I shall give an overview the roles of protein kinases in neuronal migration.

## Cytoskeleton dynamics during neuronal migration

During brain development, the extensive migratory movements of neurons from their birth place to final location are essential for neural circuit formation and proper brain function. The six-layered structure of the mammalian cerebral cortex is formed by coordinated neuronal migration *via* inside-out patterning. While early-born neurons are located in the deep layer, late-born neurons pass through the existing cortical layers to reach the superficial layer to form the six-layered structure. Three coordinated migration modes are observed in radially migrating neurons in the developing cerebral cortex: multipolar migration, glial-guided locomotion, and somal translocation (Nadarajah et al., 2001; Tabata and Nakajima, 2003; Noctor et al., 2004). During these processes, neurons change their morphology and adhesive properties. During the development of the cerebral cortex, radial migrating neurons change their morphology from multipolar to bipolar in the intermediate zone (IZ) (Tabata and Nakajima, 2003). This requires the function of cytoskeletal regulators and is inhibited by many gene mutations and experimental manipulations. These facts imply the importance of the regulation of cytoskeletal dynamics during this morphological transition. Following this, bipolar cells migrate by locomotion along the radially oriented processes of radial glia (Nadarajah et al., 2001; Noctor et al., 2004). During the mode of locomotion in migrating neurons, the nucleus is surrounded by microtubule-enriched arrays, fork-like in the front and cage-like behind (Tsai and Gleeson, 2005). Asynchronous movements of the centrosome (C) and the nucleus (N) are observed in locomotion (Tsai and Gleeson, 2005). The centrosome moves first into a cytoplasmic dilation/swelling in the leading process and then the nucleus follows (nucleokinesis) due to a pulling force from microtubules and dynein motors located at the centrosome. Cytoplasmic dilation/swelling is a structure specific to migrating neurons, at the proximal region of the leading process during the locomotion mode of migration (Nishimura et al., 2014). This coordinated relationship is called nucleus-centrosomal (N-C) coupling (Tsai and Gleeson, 2005). Retraction of trailing process occurs due to actomyosin-dependent motor functions (Bellion et al., 2005). This microtubule-actin remodeling is regulated dynamically during the locomotion mode of radial neuronal migration (Schaar and McConnell, 2005). Finally, migrating neurons along radial glial fibers change their migration mode to terminal translocation (Nadarajah et al., 2001), which is similar to somal translocation.

## Lessons from the human disorder lissencephaly

Failure of neuronal migration causes severe developmental abnormalities in the layering of the cerebral cortex and results in the human disorder lissencephaly, which means “smooth brain.” Microtubule- and actin-associated proteins regulate the dynamics of microtubule and actin cytoskeletons during neuronal migration; therefore, deletions and mutations of crucial genes involved in cytoskeletal processes lead to human lissencephaly (Dobyns, 1987) and mouse mutants with a neuronal migration phenotype.

Mutations in *doublecortin* (*DCX*) are the most common genetic cause of X-linked lissencephaly (des Portes et al., 1998; Gleeson et al., 1998). Male mice with a *Dcx* gene mutation exhibit mild histological defects only in hippocampus (Corbo et al., 2002) due to redundant compensation from *doublecortin-like kinase* (*DCLK*). This notion is supported by phenotypic analysis of *Dcx/Dclk* double-knockout (DKO) mice, which display severe abnormalities in cortical lamination due to neuronal migration defects (Deuel et al., 2006; Koizumi et al., 2006b).

**Figure 1:**
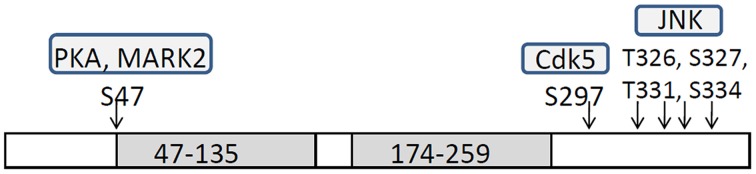
**Schematic structure of DCX and phosphorylation sites by each protein kinase**. Doublecortin (DCX) has two tubulin-binding domains, 47–135 and 174–259, and patient mutations cluster in these domains (Sapir et al., 2000; Taylor et al., 2000). DCX has S/T-P rich domain and Cdk5 and JNK phosphorylate specific sites in this domain.

DCX is a microtubule-associated protein (MAP) that has two microtubule-binding domains (Gleeson et al., 1999; Horesh et al., 1999; Taylor et al., 2000). DCX stabilizes microtubules and enhances microtubule polymerization (Francis et al., 1999; Gleeson et al., 1999; Horesh et al., 1999; Taylor et al., 2000; Moores et al., 2006). *Dcx*-deficient neurons exhibit delayed centrosomal and nuclear movements and weakened N-C coupling, indicating the involvement of DCX in these processes (Corbo et al., 2002; Koizumi et al., 2006a). DCX function is modulated by its phosphorylation by several kinases in site specific manner, including Microtubule affinity-regulating kinase 2 (MARK2), Protein kinase A (PKA), Cyclin-dependent kinase 5 (Cdk5), and c-Jun N-terminal kinases (JNKs) (Figure 1). MARK2 and PKA phosphorylate DCX at Ser47 and reduce its microtubule-binding activity (Tanaka et al., 2004a; Toriyama et al., 2012). Phosphorylation of DCX at Ser47 is also required for its proper localization to the leading process of migrating neurons (Schaar et al., 2004). Cdk5 phosphorylates DCX at Sr297 and enhances its microtubule-binding activity (Tanaka et al., 2004b). JNK phosphorylates DCX at Thr321, Thr331, and Ser334, which correspond to Thr326, Thr336, and Ser339 in mouse Dcx (Gdalyahu et al., 2004). We reported that Ser332 is also a JNK phosphorylation site of mouse Dcx (Jin et al., 2010). Phosphorylation at these sites is required for DCX localization in leading process. The importance of the balance between phosphorylation/unphosphorylation is emphasized by the requirement of a dephophorylated state of DCX at neurite tips during neuronal migration (Schaar et al., 2004).

The regulation of DCX function by phosphorylation at specific sites implicates the importance of kinase function in neuronal migration. Phosphorylation is a post-translated modification of proteins. Phosphorylation sites are categorized into two types, Tyr residues and Ser/Thr residues, which are phosphorylated by tyrosine kinases and serine/threonine kinases, respectively. The activation of Src-family tyrosine kinases by Reelin and their roles in neuronal migration will be discussed in other chapters. Thus, I will discuss the major Ser/Thr kinases that regulate neuronal migration.

## Cdk5

**Figure 2:**
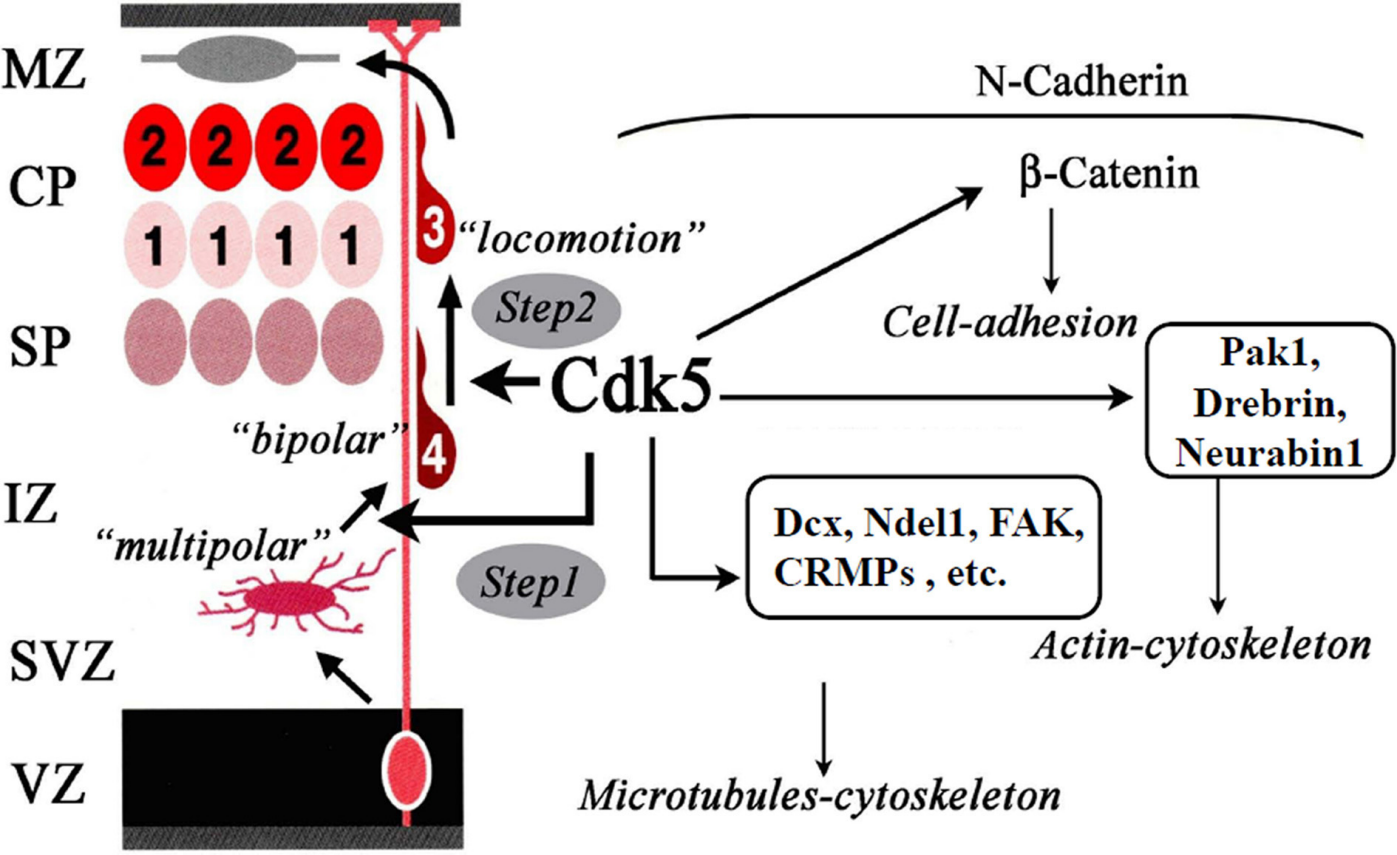
**Functions of Cdk5 in neuronal migration**. Cdk5 is required for the radial migration of later-generated neurons in the cerebral cortex. Cdk5 is necessary for multipolar-to-bipolar transition (Step 1) and locomotion through the regulation of nucleokinesis of migrating neurons (Step 2). For these steps, Cdk5 regulates the dynamics of microtubules-cytoskeleton, actin-cytoskeleton and cell-adhesion through the phosphorylation of its substrate proteins.

Cdk5 is serine/threonine kinase and its high activity is detected in post-mitotic neurons. Cdk5 forms heterodimer with its activating subunits, p35 or p39. The involvement of Cdk5 in neuronal migration was revealed by the analyses of Cdk5KO mice (Ohshima et al., 1996; Gilmore et al., 1998). Cdk5KO mice lack the laminar structure of the cerebral cortex (Ohshima et al., 1996). Birth-date labeling of the embryonic brain showed profound migration defects in cortical neurons (Gilmore et al., 1998). p35KO mice have milder abnormalities in neuronal migration (Chae et al., 1997). The identical phenotype of double-knockout p35/p39 mice and Cdk5KO mice indicates the redundant function of p35 and p39 (Ko et al., 2001). Conditional Cdk5KO mice showed an inverted cortical layer structure in layers II–VI (Ohshima et al., 2007). Cdk5 regulates multiple steps of radial migration of cortical neurons during the locomotion mode of migration (Figure 2). These include the transition from multipolar to bipolar morphology in the IZ (Ohshima et al., 2007), formation of leading processes (Kawauchi et al., 2006), and formation of a cytoplasmic dilation/swelling, which is a structure specific to migrating neurons, at the proximal region of the leading process (Nishimura et al., 2014).

Inhibition of Cdk5 activity leads to the over-stabilization of microtubules, resulting in the dysregulation of microtubule dynamics in migrating neurons (Kawauchi et al., 2005). Cdk5 phosphorylates a number of microtubule-associated proteins: DCX (Tanaka et al., 2004b), Ndel1 (Lis1-binding protein, also called Nudel) (Niethammer et al., 2000; Sasaki et al., 2000), FAK (Xie et al., 2003), and CRMP2 (Uchida et al., 2005). Ndel1 was originally identified as a novel Lis1-interacting protein and was found to be enriched at centrosomes (Niethammer et al., 2000; Sasaki et al., 2000). Ndel1 is phosphorylated by Cdk5 (Niethammer et al., 2000; Sasaki et al., 2000). Phosphorylated-Ndel1 (p-Ndel1) binds to cytoplasmic dynein heavy chain (CDHC) and katanin; its binding is required for the localization of katanin in the centrosome (Toyo-Oka et al., 2005). 14-3-3epsilon (YWHAE) binds to p-Ndel1 and protects p-Ndel1 from phosphatase attack (Toyo-Oka et al., 2003). Lis1 and 14-3-3epsilon (YWHAE) are important for neuronal migration and their deletions have been found in lissencephaly patients (Hirotsune et al., 1998; Toyo-Oka et al., 2003). These protein localizations in the centrosome, with the Lis1-Ndel1-dynein complex, regulate nucleokinesis by promoting N-C coupling during the locomotion mode of neuronal migration (Shu et al., 2004; Tsai and Gleeson, 2005). FAK phosphorylation by Cdk5 is also required for nucleokinesis (Xie et al., 2003; Xie and Tsai, 2004). CRMP2 was originally identified as an intracellular mediator of Sema3A signaling (Goshima et al., 1995). We have identified CRMP2 as a Cdk5 substrate by using Cdk5KO mouse brains (Uchida et al., 2005). Interestingly, Cdk5 phosphorylates CRMP2 at Ser522 and its phosphorylation is required for further phosphorylation of CRMP2 by GSK3β at Ser518, Thr514, and Thr509 (Uchida et al., 2005; Yoshimura et al., 2005). CRMP2 binds to the tubulin heterodimer (Fukata et al., 2002) and their binding is regulated by Cdk5/Gsk3β phosphorylation (Uchida et al., 2005; Yoshimura et al., 2005; Yamashita and Goshima, 2012). Involvement of CRMP2 and its phosphorylation in neuronal migration will be tested in CRMP2 mutant mice (Yamashita et al., 2012).

Recently, Nishimura et al. demonstrated that p27^kip1^ that is phosphorylated and stabilized by Cdk5 is required for the formation of a cytoplasmic dilation/swelling (Nishimura et al., 2014). Stabilization of p27kip1 by Cdk5 is also involved in the regulation of the actin cytoskeleton during neuronal migration (Kawauchi et al., 2006). Cdk5 phosphorylates the actin-binding proteins, Drebrin and Neurabin-I, and may regulate neuronal migration (Causeret et al., 2007; Tanabe et al., 2014).

**Figure 3:**
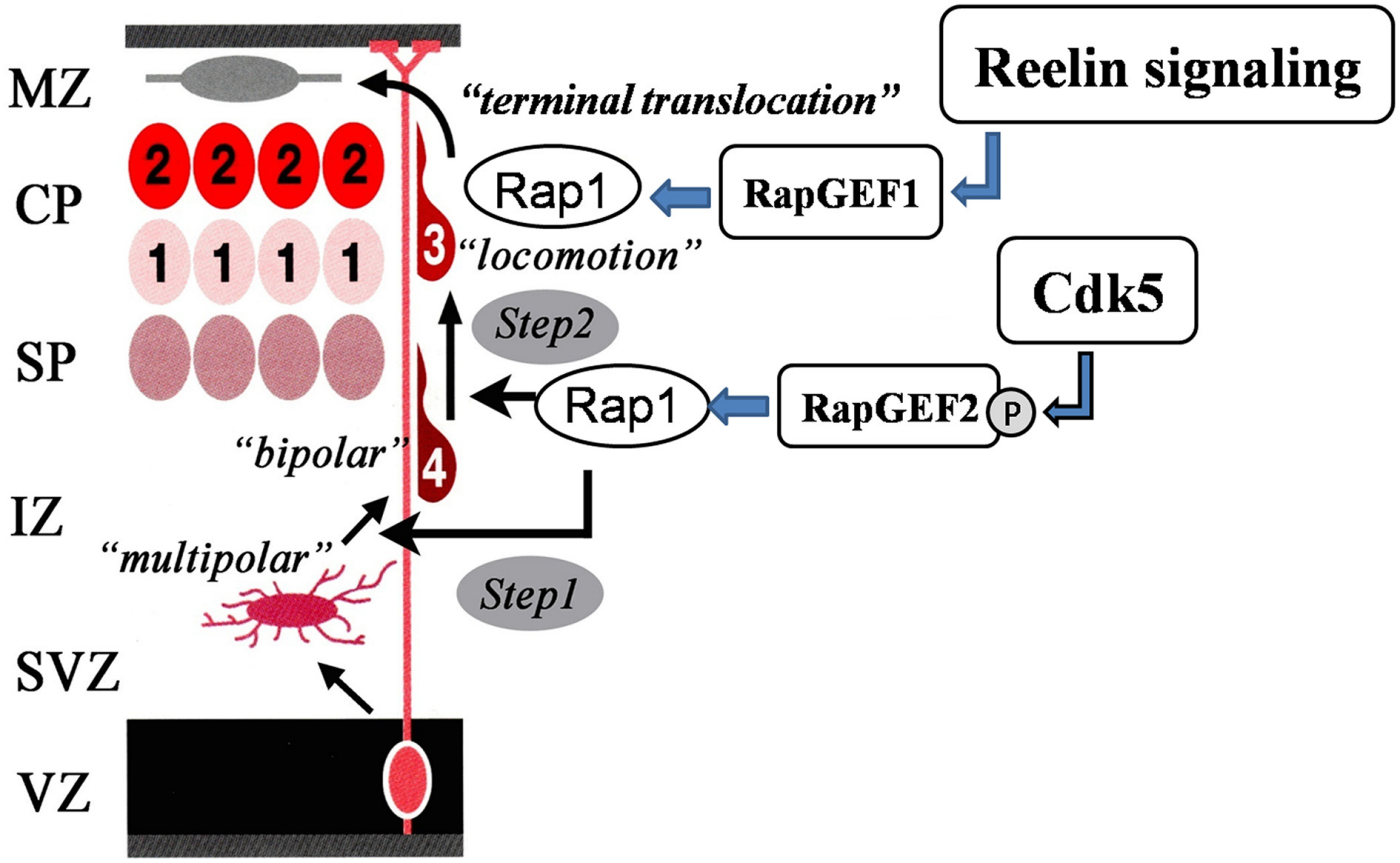
**Sequential Rap1 activation by Cdk5 and Reelin signaling**. Cdk5 and Reelin signaling activate Rap1 through the activation of different Rap1GEFs in the control of the radial migration of cortical neurons in the cerebral cortex in a sequential manner.

Rap1 signaling is involved in neuronal migration and is regulated by Cdk5 (Utreras et al., 2013). Rap1 activation promotes the cell-surface localization of N-cadherin (Jossin and Cooper, 2011). The N-cadherin-mediated adhesion complex is required for multipolar-bipolar transition (Jossin and Cooper, 2011) and radial fiber-dependent neuronal migration (Kawauchi et al., 2010). A previous study has shown that pharmacological inhibition of Cdk5 activity enhances N-cadherin-mediated cell-cell adhesion (Kwon et al., 2000). Rap1 activation depends upon Rap1-GEFs, including Rap1GEF1 (also known as C3G) and Rap1GEF2. RapGEF1 activation of Rap1 controls somal/terminal translocation triggered by Reelin (Franco et al., 2011; Jossin and Cooper, 2011; Sekine et al., 2012) *via* the stabilization of leading processes toward the marginal zone (Franco et al., 2011; Sekine et al., 2012). Interestingly, RapGEF2 KO mice showed a neuronal migration defect phenotype in the subcortical area, which indicated the involvement of RapGEF2 in multipolar-bipolar transition (Bilasy et al., 2009). Recently, Ye et al. have shown that Cdk5 phosphorylates RapGEF2 at Ser1124 and its phosphorylation is required for Rap1 activation (Ye et al., 2014). Previous studies have shown that RapGEF1-dependent Rap1 activation is dispensable in multipolar-bipolar transition (Sekine et al., 2012); therefore, Cdk5 mediated Rap1 activation *via* RapGEF2 phosphorylation is important for this transition. As proposed by Ye et al. (2014), the two pathways of Reelin and Cdk5 are not simply parallel, but rather act on successive phases of neuronal migration *via* Rap1 activation. Cdk5-mediated RapGEF2 phosphorylation controls multipolar-bipolar transition and Reelin-mediated RapGEF1 activation promotes terminal translocation (Figure 3). This idea fits well with our previous observations in mutant mice that lack Cdk5/p35 and Reelin/Dab1 (Ohshima et al., 2001, 2002; Ohshima and Mikoshiba, 2002).

Cdk5 is also required for the radial migration of hippocampal neurons (Ohshima et al., 1996, 2007) and Purkinje cells in the developing cerebellum (Ohshima et al., 1999; Kumazawa et al., 2013). Inward migration of granule cells and migration in the rostral migratory stream is also Cdk5-dependent (Ohshima et al., 1999; Hirota et al., 2012; Kumazawa et al., 2013; Umeshima and Kengaku, 2013). Compared with the analysis of the molecular mechanisms of neuronal migration in radial migration in the cerebral cortex, the mechanisms of neuronal migration in hippocampal and cerebellar neurons remain to be elucidated.

## GSK3β

Two members of the GSK-3 family in mammals, GSK3α and GSK3β, show 98% amino acid sequence identity within their kinase domains and overall share 85% identity (Doble and Woodgett, 2003). Both isoforms are highly expressed in the developing brain. GSK3-signaling is a strong regulator of neuronal progenitor proliferation in the developing cerebral cortex (Chenn and Walsh, 2002; Kim et al., 2009). To study the role of GSK3 in neuronal migration, Morgan-Smith et al. produced *Gsk3a*^−/−^*Gsk3b*^*loxP*/*loxP*^; *Neurod6-Cre* (*Gsk3:Neurod6*) mice and analyzed neuronal positioning after birth. The *Nuerod6-Cre* mice induce recombination in post-mitotic cortical excitatory neurons after E11 (Goebbels et al., 2006). *Gsk3*-deleted neurons expressing the upper layer marker exhibited migration failure in the cerebral cortex. Radial migration in the hippocampus was also affected (Morgan-Smith et al., 2014). Hypophosphorylation of CRMP2 at Thr514 (Yoshimura et al., 2005) and Dcx at Ser327 (Bilimoria et al., 2010) was observed in the cortex of *Gsk3:Neurod6* mice (Morgan-Smith et al., 2014).

## JNK

JNKs are members of MAPK signaling pathway. There are three related genes in mammals: *Jnk1, Jnk2, and Jnk3*. All three *Jnk* genes are expressed in the developing mouse brain. JNKs act as the final effector kinases within a classical cascade consisting of MAPKKKs (MAP3Ks), MAPKKs (MAP2Ks), and MAPKs. Like other MAPKs, JNKs are activated by MAP2K-mediated phosphorylation. MKK4 and MKK7 are the MAP2Ks that phosphorylate JNKs.

Genetic deletion studies of *Jnk1* and the MAP3K and MAP2Ks for Jnk1, *Dlk1, Mkk4*, and *Mkk7*, in mice suggest their involvements in the migration of cortical projection neurons (Hirai et al., 2006; Wang et al., 2007; Westerlund et al., 2011; Yamasaki et al., 2011). Deletion of the upstream activators of JNKs, *Dlk1* (Hirai et al., 2006), *Mkk4* (Wang et al., 2007), and *Mkk7* (Yamasaki et al., 2011) inhibits radial migration. On the other hand, deletion of *Jnk1* results in accelerating radial migration (Westerlund et al., 2011). These results could be explained by *Jnk2* and/or *Jnk3* playing opposing roles to *Jnk1* in radial migration. Double deletion of *Jnk1* and *Jnk2* causes embryonic lethality (Kwon et al., 2000); therefore, further study using the conditional deletion of genes will be necessary to resolve this issue. Pharmacological inhibition of JNK activity using SP600125 inhibits the radial migration of cortical neurons (Kawauchi et al., 2003; Hirai et al., 2006). However, a recent study has shown that SP600125 inhibits 74 kinases (out of 353 tested) at 10 μM, including MEK1, MEK2, MKK3, MKK4, and MKK6 (KINOMEscan LINCS data base). Thus, the results obtained using SP600125 are difficult to interpret because of its low specificity for JNK.

JNKs phosphorylate the microtubule regulatory proteins, DCX, MAP2, MAP1b, and SCG10 (Chang et al., 2003; Kawauchi et al., 2003; Gdalyahu et al., 2004; Tararuk et al., 2006; Jin et al., 2010; Björkblom et al., 2012). We have shown that phosphorylation of DCX at Ser332 by JNK disrupts its microtubule binding (Jin et al., 2010). SCG10 is a tubulin interacting protein, which is phosphorylated by JNK SCG10 at Ser62 and Ser73 (Tararuk et al., 2006). Phosphorylation of SCG10 at Ser73 is reduced in *Jnk1*^−/−^ brains (Tararuk et al., 2006). Knockdown of SCG10 increases the rate of radial migration (Westerlund et al., 2011), suggesting a role for SCG10 in neuronal migration. The involvement of JNK in the regulation of the tangential migration of inhibitory neurons from ganglionic eminence is also reported (Myers et al., 2014).

## MARK2

MARK2/Par-1 was originally identified as a regulator of cell polarity in *C. elegans* (Par-1). In parallel it was also identified as a protein kinase that regulates microtubule stability, microtubule affinity-regulating kinase 2 (MARK2) (Drewes et al., 1997). *In vivo* overexpression of MARK2/Par-1 results in a loss of neuronal polarity (Sapir et al., 2008). A reduction in MARK2/Par-1 causes neuronal migration arrest with more stable microtubules (Sapir et al., 2008). MARK2/Par-1 phosphorylates tau, MAP2, MAP4, and DCX (Biernat et al., 1993; Drewes et al., 1997; Schaar et al., 2004). Phosphorylation of these microtubule-associated proteins (MAPs) causes the removal of MAPs and DCX from microtubules.

## shRNA-mediated off-target toxicity causes neuronal migration defects

Acute inactivation of gene function by shRNA, together with in utero electroporation, is a widely used method to study neuronal migration. In some cases, such as DCX, neuronal migration phenotypes caused by shRNA knockdown or knockout by gene deletion show a discrepancy (Corbo et al., 2002; Bai et al., 2003). Recently, Baek et al. have shown that shRNAs cause neuronal migration defects *via* an off-target effect (Baek et al., 2014). They have demonstrated that shRNA alters endogenous miRNA pathways and leads to reduced let7 miRNA expression. This disruption of let7 causes neuronal migration defects. They have designed scrambled shRNAs of *Dcx* and found half cause neuronal migration defects. These results offer a warning for the interpretation of neuronal migration studies using shRNAs. They have also shown that switching from shRNA to a shmiRNA construct can avoid these toxic effects. Therefore, studies of neuronal migration using the shRNA method need to be re-evaluated by knockdown studies using shmiRNA or genetic deletion.

## Future prospects of research

The activation of protein kinases are regulated by intrinsic and extrinsic factors. For example, Cdk5 activity is regulated by the amount of its activating subunits, p35 and p39. p35, and p39 are expressed in post-mitotic neurons; therefore, they are regulated by the degree of neuronal maturation. Cdk5 activity is also regulated by several extracellular factors (Sasaki et al., 2002; Cheung et al., 2007; Fu and Ip, 2007; Fu et al., 2007). Gsk3β activity is regulated by Wnt signaling and JNK activity is regulated by extracellular stimuli. Therefore, coordinated neuronal migration is regulated by multiple signaling pathways external to migrating neurons through the balanced activation of protein kinases as discussed above. One direction for future studies will be to examine the molecular mechanisms that regulate protein kinase activity by extracellular factors. For example, Sema3A is shown to regulate radial migration (Chen et al., 2008); however, its regulation of intracellular protein kinase activity remains to be elucidated. For this purpose, the development of a method to monitor kinase activity *in vivo* will be valuable for the future research. Studies on the identification of the downstream effectors (substrates) of protein kinases are important to understand the mechanisms by which each protein kinase is involved in neuronal migration. In this regard, comparative phosphoproteomics using brain samples from kinase-null mutant mice will be useful (Uchida et al., 2005; Contreras-Vallejos et al., 2014).

### Conflict of interest statement

The author declares that the research was conducted in the absence of any commercial or financial relationships that could be construed as a potential conflict of interest.
